# Optical Coherence Tomography Assessment of Tongue Papillary Atrophy and Patient-Reported Impact on Taste Following Head and Neck Cancer Therapy

**DOI:** 10.3390/jcm15041577

**Published:** 2026-02-17

**Authors:** Zaid Hamdoon, Waseem Jerjes, Dara Rashed, Colin Hopper

**Affiliations:** 1 College of Dental Medicine, University of Sharjah, Sharjah 27272, United Arab Emirates; 2 Unit of OMFS, UCL Eastman Dental Institute, London WC1E 6DE, UK; d.rashed@alumni.ucl.ac.uk (D.R.); c.hopper@ucl.ac.uk (C.H.); 3 Faculty of Medicine, Imperial College London, London W12 0BZ, UK

**Keywords:** tongue, taste disorders, optical coherence tomography, chemotherapy, radiotherapy, xerostomia

## Abstract

**Background**: Taste disturbances are common after head and neck cancer treatment and can impair nutrition and quality of life. Aim: The aim of this study was to characterise tongue papilla morphology using optical coherence tomography (OCT), apply an ordinal atrophy grading system, and evaluate associations between structural changes and patient-reported taste outcomes. **Materials and Methods**: A case–control study was conducted including 53 participants: 33 head and neck cancer survivors (radiotherapy, chemotherapy, or combined treatment) and 20 healthy controls. OCT was used to assess papilla height, width, and signal intensity, and a five-point ordinal atrophy scale was applied to fungiform and filiform papillae. Taste outcomes were assessed using the UW-QOL (v4). Interobserver reliability was evaluated using Cohen’s kappa. **Results**: Cancer patients exhibited significantly reduced papilla height and width compared with controls (fungiform mean difference −350 µm; filiform −250 µm; all *p* < 0.001). Radiotherapy and combined therapy were associated with more severe atrophy than chemotherapy alone. Severe fungiform atrophy was strongly associated with patient-reported taste impact (UW-QOL taste domain; Spearman ρ = 0.80), with up to 90% reporting impairment at the highest atrophy grades. After controlling for age, the association attenuated but remained statistically significant (partial Spearman ρ = 0.39, *p* = 0.04; regression β = 0.14, 95% CI 0.03–0.25, *p* = 0.01), and it was further attenuated after additional adjustment for xerostomia (UW-QOL saliva domain; β = 0.09, 95% CI −0.02–0.20, *p* = 0.11). Interobserver agreement was substantial. **Conclusions**: OCT enables non-invasive, high-resolution assessment of tongue papillary atrophy and its cross-sectional association with patient-reported taste impact after head and neck cancer therapy. Findings should be interpreted as associative; age-matched and longitudinal studies incorporating objective taste testing are warranted.

## 1. Introduction

Taste disturbances—including ageusia, dysgeusia and hypogeusia—are among the most frequent toxicities experienced by people treated for head and neck cancer. During radiotherapy, approximately 60–95% of patients report clinically meaningful taste change, and a substantial minority continue to report impairment months to years after treatment, particularly when the anterior tongue receives a higher radiation dose. These symptoms can reduce oral intake, contribute to weight loss and malnutrition risk, and are consistently associated with poorer quality of life; yet they remain under-recognised in routine follow-up and variably assessed in practice [1,2,3,4].

While subjective taste alterations are commonly reported, the underlying biological changes remain insufficiently characterised. One key contributor is papillary atrophy, particularly of the fungiform papillae, which house a substantial portion of taste buds [5,6]. Taste dysfunction is likely multifactorial, reflecting direct injury to taste-bud cells and their progenitor niche as well as treatment-related xerostomia and mucosal inflammation, which together may amplify both acute dysgeusia and longer-term impairment [5,6,7,8,9]. However, the extent and morphology of such atrophy are rarely quantified systematically, partly due to the limitations of current diagnostic tools. Conventional assessments rely heavily on visual inspection or indirect measures, which are prone to variability and often insensitive to subtle structural changes [7,8,9,10,11,12].

Recent advances in Optical Coherence Tomography (OCT) offer a promising non-invasive solution for high-resolution visualisation of superficial oral structures. OCT enables in vivo cross-sectional imaging of epithelial and subepithelial layers, making it well-suited for studying tongue papillae [13,14,15,16,17,18]. While OCT has previously been explored in the context of oral mucositis and squamous cell carcinoma, its application to tongue papillae morphology and taste dysfunction remains largely unexplored in human subjects [19,20,21,22,23,24,25].

This study addresses this gap by applying OCT to characterise fungiform and filiform papillae in cancer patients and healthy controls [15,16,26,27]. A novel five-point atrophic scoring system was developed and applied to standardise assessment across subjects. Unlike previous approaches that focus solely on descriptive morphology, this grading system integrates papilla height, width, and signal reflectivity—parameters easily captured by OCT.

The primary aim was to determine whether papillary atrophy, as measured by OCT, correlates with patient-reported taste impact in patients undergoing cancer therapy. The study also sought to explore differences in atrophic severity between treatment modalities (radiotherapy vs. chemotherapy vs. combined), and to validate the reproducibility of the scoring system through interobserver agreement.

By combining OCT-derived structural imaging with patient-reported taste impact, this study provides an approach to characterising papillary morphology after head and neck cancer therapy and to exploring associations between tissue changes and perceived taste-related quality of life.

## 2. Material and Methods

### 2.1. Study Design and Participants

This was a case–control study designed to characterise morphological changes in tongue papillae using OCT by comparing patients previously treated for head and neck cancer (cases) with healthy participants (controls).

A total of 53 participants were enrolled: 33 patients previously treated for head and neck cancer and 20 healthy controls. Cancer patients were stratified into three subgroups based on treatment exposure—radiotherapy only (n = 12), chemotherapy only (n = 11), and combined radiotherapy and chemotherapy (n = 10)—reflecting standard treatment pathways in contemporary head and neck cancer management [28,29]. Patients were recruited from the Head and Neck Department at University College London Hospitals.

Healthy controls, aged between 20 and 36 years, were recruited via local advertisement using convenience sampling to provide normative OCT reference measurements for tongue papillae morphology. They were screened to ensure absence of cancer history, chronic illness, or oral/systemic conditions known to affect mucosal health or taste. We recognise that this control sampling strategy may introduce selection bias and may not be age-matched to the cancer cohort; this limitation is addressed explicitly in the Discussion.

### 2.2. Ethical Approval and Consent

The study was conducted in accordance with the Declaration of Helsinki and received approval from the Moorfields & Whittington Local Research Ethics Committee (REC reference: 07/Q0504/4). Written informed consent was obtained from all participants after they were fully briefed on the study’s aims, procedures, and potential risks. All data were anonymised at the point of collection and stored securely on password-protected institutional servers.

### 2.3. Inclusion and Exclusion Criteria

Participants in the cancer group were eligible if they were adults (aged ≥ 18 years) with a confirmed diagnosis of head and neck cancer who had previously received radiotherapy and/or systemic anticancer therapy (e.g., platinum-based chemotherapy), consistent with standard contemporary treatment pathways in head and neck oncology. Treatment modality (radiotherapy only, chemotherapy only, or combined chemoradiotherapy) was recorded for subgroup analyses [28,29]. Taste disturbance was not an eligibility criterion; instead, patient-reported taste and saliva outcomes were captured using the University of Washington Quality of Life questionnaire UW-QOL (version 4) as described below [30,31].

Healthy control participants were eligible if they had no history of head and neck cancer or prior radiotherapy/chemotherapy, no current oral pathology on clinical inspection, and no chronic illness or regular medication known to materially affect taste perception or oral mucosal integrity (e.g., commonly reported causes of drug-related dysgeusia) [32].

Exclusion criteria for all participants included active oral infections, mucosal lesions, recent oral surgery, autoimmune conditions affecting the oral cavity, and congenital tongue disorders. Individuals unable to provide informed consent, were also excluded to minimise confounding and ensure study safety and reliability.

### 2.4. Data Collection

All data were collected during a single outpatient visit at University College London Hospitals. Upon arrival, participants completed a structured intake interview to document demographic details, medical history, cancer treatment information (if applicable), and lifestyle factors including tobacco and alcohol use.

Following the interview, participants completed the UW-QOL, a validated head and neck cancer-specific patient-reported outcome instrument that includes taste and saliva domains relevant to oral sensory function [30,31]. The questionnaire was administered prior to imaging to avoid potential bias introduced by the examination process.

Subsequently, OCT imaging was conducted in a dedicated clinical suite under controlled lighting. The imaging procedure typically lasted 15–20 min and was performed by trained operators following a standardised protocol consistent with established approaches for in vivo OCT imaging of oral mucosa [33,34,35]. All acquired OCT scans were anonymised immediately after capture and archived securely. Data processing and analysis were performed in batches to preserve observer blinding and reduce intra-session bias.

### 2.5. Taste Disturbance Assessment

Taste-related patient-reported outcomes were assessed using the UW-QOL, a validated head and neck cancer-specific instrument that includes dedicated domains for taste and saliva. Participants completed the UW-QOL prior to OCT imaging. For the present analyses, the taste domain was used as the primary patient-reported taste outcome, and the saliva domain was considered as a relevant co-symptom given its potential contribution to perceived taste impairment [30,31]. Severity was rated on a five-point scale ranging from 1 (no disturbance) to 5 (severe disturbance affecting daily life).

In addition to the UW-QOL domains, participants completed a brief set of study-specific questions addressing specific taste qualities (sweet, sour, salty, bitter and umami), the temporal onset of symptoms in relation to treatment, and associated oral symptoms (e.g., dry mouth or burning sensation), reflecting commonly reported symptom patterns following head and neck cancer therapy [36]. Participants completed the questionnaire before undergoing OCT imaging to ensure responses were uninfluenced by the examination process. Responses were later analysed in conjunction with morphological imaging data to explore associations between taste disturbance severity and papillary atrophy scores.

### 2.6. Optical Coherence Tomography (OCT) Imaging

All participants underwent in vivo imaging of the dorsal tongue surface using a commercially available anterior-segment optical coherence tomography (OCT) system (spectral-domain OCT), configured with a 2.0 mm telecentric lens.

Imaging was focused on the anterior two-thirds of the tongue, capturing areas rich in fungiform and filiform papillae [36,37]. During the procedure, participants were seated upright and asked to gently protrude their tongue, which was then stabilised using sterile gauze to minimize movement artefact. Cross-sectional OCT scans were acquired with a resolution of 6 µm axially and 15 µm laterally, and a penetration depth of 2 mm [33,34,35]. Each scan lasted under five seconds to ensure participant comfort and reduce motion artefact.

A total of five representative scans were obtained per participant, ensuring adequate capture of both papillae types, in keeping with established in vivo oral OCT acquisition approaches [33,34,35]. Images were saved in a lossless format and anonymised at the point of capture to ensure subsequent blinded evaluation.

### 2.7. Observer Training and Image Scoring

Two independent observers, blinded to participants’ group allocation and questionnaire responses, conducted the evaluation of all OCT images. Prior to formal scoring, both observers underwent structured training using a reference set of 30 anonymised OCT scans representing the full spectrum of observed papillary morphology. Training involved supervised calibration sessions to standardise the use of grading criteria and ensure interobserver consistency.

Each image was assessed for two papillae types—fungiform and filiform—using a newly developed five-point ordinal atrophic grading scale. The scale was defined a priori through consensus between the study investigators based on established OCT structural descriptors used in oral mucosal imaging (including papilla height, epithelial thickness, and internal reflectivity patterns) [33,34,35]. A score of 1 indicated normal morphology, while a score of 5 represented severe atrophy with architectural loss.

The ordinal grading framework was designed not only to standardise structural OCT assessment but also to allow consistent alignment between morphological features and patient-reported taste outcomes. Accordingly, Table 1 presents the predefined atrophic score categories and their corresponding OCT morphological descriptors. The inclusion of patient-reported taste categories reflects the study’s integrated structural–symptom framework and does not imply a direct causal relationship between papillary morphology and functional taste impairment.

For each participant, three to five scans were evaluated, and the median score for each papilla type was recorded. Interobserver agreement was calculated using Cohen’s Kappa (κ) statistic. Any score discrepancies greater than one point between observers were reviewed jointly, and consensus was reached through a third independent review where necessary.

### 2.8. Quantitative Morphometric Analysis

In addition to ordinal atrophy scoring, quantitative morphometric analysis was performed on the OCT images to objectively assess structural differences in tongue papillae. Using calliper and measurement tools embedded within the imaging software, the following parameters were manually measured for both fungiform and filiform papillae—consistent with established OCT-based structural assessment of oral mucosa: papilla height (vertical distance from base to apex), base width (horizontal span at the epithelial surface), epithelial thickness, and internal reflectivity patterns [33,34,35].

Measurements were taken from the most representative cross-sectional slice of each papilla, selected based on clarity and alignment. At least three measurements per papilla type were obtained per participant, and mean values were calculated for statistical analysis.

These quantitative metrics were compared across treatment subgroups and controls to identify morphological differences associated with cancer therapy. Correlation analyses were also performed between morphometric values and both taste disturbance scores and ordinal atrophy grades.

### 2.9. Demographic and Lifestyle Variables

Demographic and lifestyle information was collected at enrolment using a structured, study-specific intake form administered as a standardised interview. Data included age, gender, smoking status (never, former, or current), and alcohol consumption patterns.

Among the 33 participants in the cancer group, 23 were male and 10 were female, with an age range of 40 to 55 years. Within this group, 36% were classified as former smokers, and 18% were current smokers at the time of participation. Alcohol use was reported by 62% of cancer patients, with 21% consuming more than 14 units per week.

The control group included 8 males and 12 females, aged between 20 and 36 years (mean age: 29 years). Occasional smoking was reported by 5% of controls, while 30% reported alcohol use, with an average intake of 7 units per week. These variables were included in the final analysis as potential confounders in the relationship between treatment exposure, papillary morphology, and patient-reported taste impact.

### 2.10. Statistical Analysis

Statistical analyses were performed using SPSS Statistics version 28.0 (IBM Corp., Armonk, NY, USA). Descriptive statistics were used to summarise participant characteristics and morphometric data. Continuous variables were expressed as means ± standard deviations, and categorical variables as frequencies and percentages.

Normality of continuous variables was assessed using visual inspection of histograms and Q–Q plots and the Shapiro–Wilk test. Homogeneity of variance was evaluated using Levene’s test. Where assumptions for parametric testing were not met, non-parametric alternatives were used (Mann–Whitney U test for two-group comparisons and Kruskal–Wallis test for three-group comparisons), with Dunn–Bonferroni adjustment for post hoc analyses.

Group comparisons for papilla height, base width, and atrophy scores were conducted using one-way ANOVA with Tukey’s post hoc test for multiple comparisons. Independent *t*-tests were used for direct comparisons between the cancer and control groups when appropriate.

Interobserver agreement for ordinal scoring was assessed using Cohen’s Kappa (κ) statistic. Spearman’s rank correlation coefficient (ρ) was used to explore associations between patient-reported taste outcomes (UW-QOL taste domain) and both ordinal atrophy scores and quantitative papillary measurements.

A *p*-value of less than 0.05 was considered statistically significant for all tests.

To address potential confounding by age, additional age-adjusted analyses were conducted for key structure–symptom relationships. Partial Spearman correlations were calculated between UW-QOL taste scores and OCT papillary measures while controlling for age. In addition, linear regression models were fitted with papilla height as the dependent variable and UW-QOL taste score and age as covariates to estimate associations independent of age. To assess the influence of shared symptom burden inherent to self-reported outcomes, a sensitivity model was performed by additionally adjusting for UW-QOL saliva (xerostomia) scores. All variables included in these models were complete for the analysed sample.

## 3. Results

Fungiform papillae exhibited significant morphological differences between cancer patients and healthy controls across all measured parameters. In the cancer group, the average height of fungiform papillae was markedly reduced, ranging from 150 to 400 µm, with a mean of 250 ± 50 µm. In contrast, the healthy control group had taller papillae ranging from 400 to 750 µm, with a mean of 600 ± 100 µm (*p* < 0.001).

A similar trend was observed for papillary width. Cancer patients demonstrated a mean width of 250 ± 40 µm, significantly narrower than the mean width of 400 ± 50 µm observed in controls (*p* < 0.001). These quantitative reductions in papilla size were accompanied by elevated atrophic scores; most cancer patients scored between Grades 3 and 4, indicating moderate to severe atrophy, whereas healthy controls consistently received scores of 1, reflecting normal morphology.

Morphological alterations included flattening of the papillae and pronounced reductions in vascular signal visibility, as captured on OCT imaging. Additionally, internal reflectivity within the papillae was significantly lower in cancer patients, consistent with structural degradation, whereas healthy controls displayed uniformly high reflectivity (*p* < 0.001).

Consistent with these structural differences, patient-reported taste impact (UW-QOL) was more prevalent in the cancer cohort (65%) than in healthy controls (0%) (*p* < 0.001). The observed association between fungiform atrophy and UW-QOL taste scores should be interpreted cautiously because taste disturbance was assessed by self-report and may reflect perceived taste changes influenced by co-occurring symptoms (e.g., xerostomia) rather than objective gustatory dysfunction alone.

Effect size calculations further highlighted the magnitude of these differences. For fungiform papilla height, the effect size was 1.45, and for width, it was 1.20—both considered large, indicating clinically and statistically significant deviations.

Filiform papillae also demonstrated significant morphological degradation in cancer patients compared to healthy controls. In the cancer group, filiform papillae exhibited a height range of 10 to 300 µm, with a mean of 150 ± 40 µm. This was markedly lower than in the control group, where heights ranged from 250 to 500 µm, with a mean of 400 ± 80 µm (*p* < 0.001).

The average width of filiform papillae was similarly reduced in cancer patients, with a mean of 120 ± 30 µm, in contrast to 200 ± 40 µm in healthy individuals (*p* < 0.001). These structural differences were reflected in the atrophic scoring, where cancer patients most frequently received scores of 3 or higher—indicative of moderate to severe atrophy. In contrast, healthy controls uniformly received scores of 1, signifying normal morphology.

OCT imaging revealed distinctive changes in filiform architecture among cancer patients, including loss of keratinised projections, irregular contours, and the emergence of short, stub-like remnants in advanced cases. Internal reflectivity was significantly diminished in the cancer group, consistent with keratin depletion and tissue disorganisation. Conversely, healthy controls displayed strong, uniform reflectivity and intact filiform structure (*p* < 0.001).

Filiform papillae are non-gustatory; in this cohort, higher filiform atrophy grades co-occurred with poorer UW-QOL taste scores, which may reflect overall treatment-related oral morbidity, but oral comfort/texture perception and broader mucosal injury were not measured and therefore functional implications should not be inferred.

**Figure 1:**
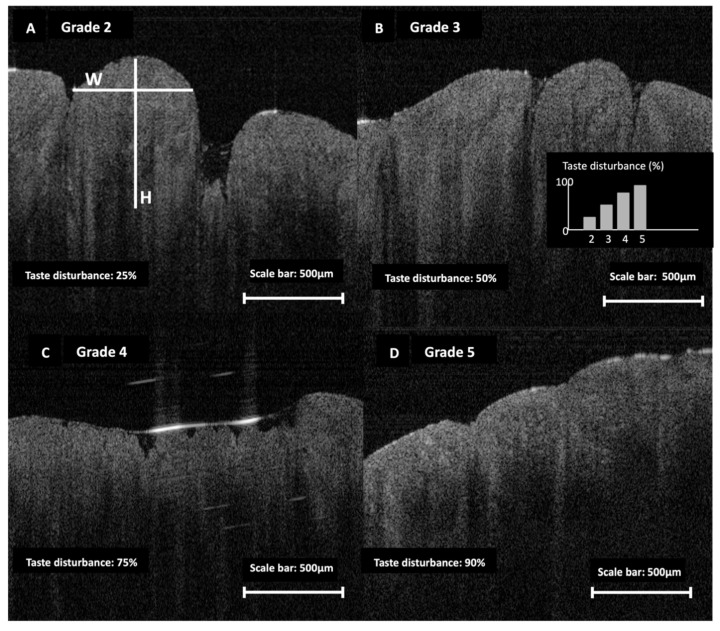
OCT-based ordinal staging of fungiform papillary atrophy and associated taste disturbance. Representative OCT B-scans illustrate Grades 2–5 (**A**–**D**) as defined by the study’s fungiform atrophy scale. Panel A shows example morphometric measurements (H, papilla height; W, base width). Taste disturbance (%) denotes the proportion reporting impairment on the UW-QOL taste domain within each atrophy grade (inset). Scale bars are shown in each panel.

Quantitative analysis confirmed significant morphological differences in both fungiform and filiform papillae between cancer patients and healthy controls. The mean height difference for fungiform papillae was −350 µm, with a 95% confidence interval (CI) ranging from −380 to −320 µm (*p* < 0.001). The corresponding effect size was 1.45, indicating a large and clinically meaningful difference. For fungiform papillary width, the mean difference was −150 µm [95% CI: −180 to −120 µm], also significant at *p* < 0.001, with an effect size of 1.20. Representative OCT B-scans illustrate the progressive fungiform papillary atrophy captured by the ordinal grading system (Grades 2–5). Higher atrophy grades were associated with a greater proportion of participants reporting taste disturbance on the UW-QOL taste domain (Figure 1).

**Figure 2:**
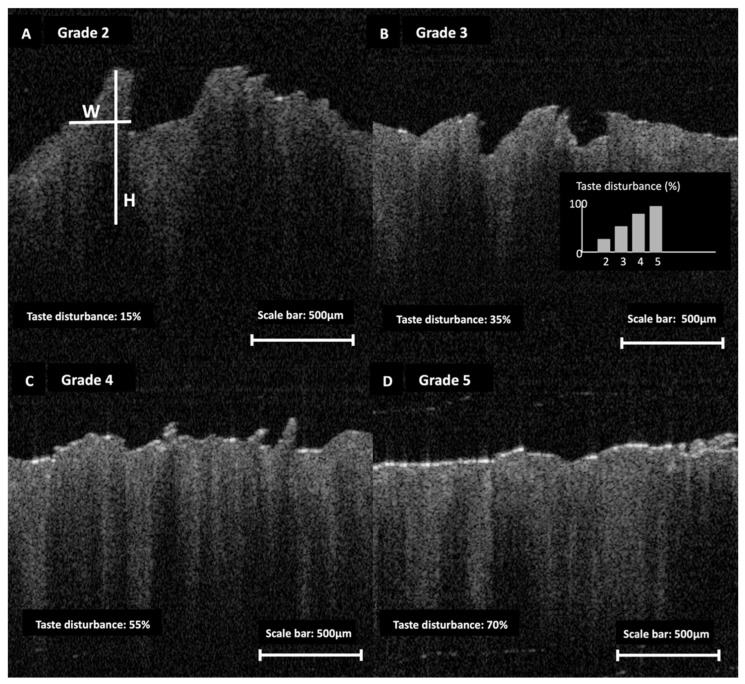
OCT-based ordinal staging of filiform papillary atrophy and associated taste disturbance. Representative OCT B-scans illustrate Grades 2–5 (**A**–**D**) of the study’s filiform atrophy scale. Panel A shows example morphometric measurements (H, papilla height; W, base width) obtained using the software calliper tool. Taste disturbance (%) denotes the proportion reporting impairment on the UW-QOL taste domain within each filiform atrophy grade (inset). Scale bars are shown in each panel.

In the case of filiform papillae, the mean height difference was −250 µm [95% CI: −280 to −220 µm, *p* < 0.001], corresponding to an effect size of 1.30. The mean width difference was −80 µm [95% CI: −100 to −60 µm, *p* < 0.001], with an effect size of 0.90. Filiform papillae also demonstrated progressive surface simplification across increasing atrophy grades on OCT. Higher filiform atrophy grades were accompanied by a greater proportion of participants reporting UW-QOL taste disturbance (Figure 2).

**Figure 3:**
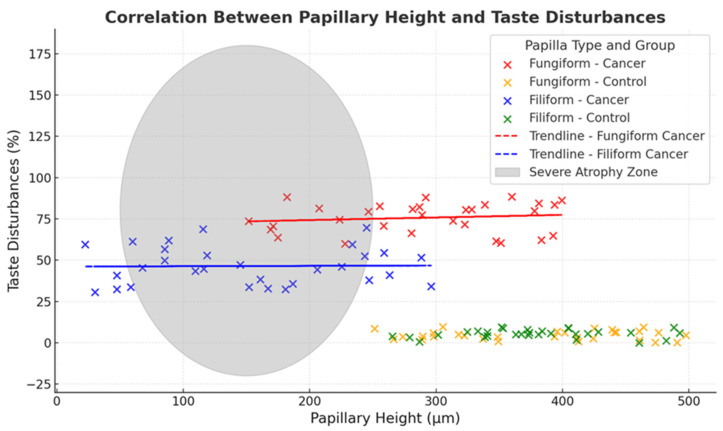
Association Between Papillary Height and Patient-Reported Taste Impact (UW-QOL) in Cancer Patients and Healthy Controls. This figure shows the relationship between papillary height and reporting of taste impact on the UW-QOL taste domain for fungiform and filiform papillae. In cancer patients, lower papillary height co-occurred with a higher prevalence of reported taste impact, particularly in the radiotherapy group. Healthy controls exhibited higher papillary heights and no reported UW-QOL taste impact. These findings reflect cross-sectional associations based on patient-reported outcomes and do not establish mechanistic or causal relationships.

Interobserver agreement in atrophic grading was high. Cohen’s Kappa (κ) statistic was 0.76 for fungiform papillae and 0.71 for filiform papillae, indicating substantial agreement and supporting the reliability of the scoring method. These results demonstrate consistent measurement and robust differentiation between study groups based on OCT-derived morphometric and ordinal data. Quantitative differences in papillae height and width between cancer and control groups are detailed in Table 2. Interobserver agreement statistics are presented in Figure 3, demonstrating high scoring consistency.

A strong unadjusted association was observed between fungiform papillary atrophy and patient-reported taste impact among cancer patients. Specifically, fungiform papilla height was associated with the UW-QOL taste domain (Spearman ρ = 0.80), indicating a strong monotonic relationship between papillary morphology and perceived taste impact. Because taste outcomes were captured using a patient-reported quality-of-life measure rather than psychophysical gustatory testing, and because co-occurring oral symptoms (e.g., xerostomia) may influence perceived taste, these correlations should be interpreted cautiously.

Age-adjusted analyses were undertaken to assess whether the observed structure–symptom relationship could be explained by age differences between cohorts. After controlling for age, the association between fungiform papilla height and UW-QOL taste attenuated to a moderate magnitude but remained statistically significant (partial Spearman ρ = 0.39, *p* = 0.04). In linear regression models including age, UW-QOL taste remained independently associated with fungiform papilla height (β = 0.14, 95% CI 0.03–0.25, *p* = 0.01), indicating that age accounted for part, but not all, of the observed association. In a sensitivity model additionally adjusting for UW-QOL saliva (xerostomia), the association between fungiform height and UW-QOL taste was further attenuated and no longer statistically significant (β = 0.09, 95% CI −0.02–0.20, *p* = 0.11), consistent with overlap between perceived taste impact and broader oral symptom burden.

Patients with the highest atrophic scores—Grades 4 and 5—reported the greatest prevalence of patient-reported taste impact. Among those with Grade 5 atrophy, 90% reported significant taste impairment, while 75% of patients with Grade 4 atrophy also reported moderate to severe patient-reported taste impact. Conversely, patients with lower scores showed lower rates of taste disturbance: 50% of those with Grade 3 and 25% of those with Grade 2 reported altered taste. No disturbances were reported by participants with a normal fungiform morphology (Grade 1).

**Figure 4:**
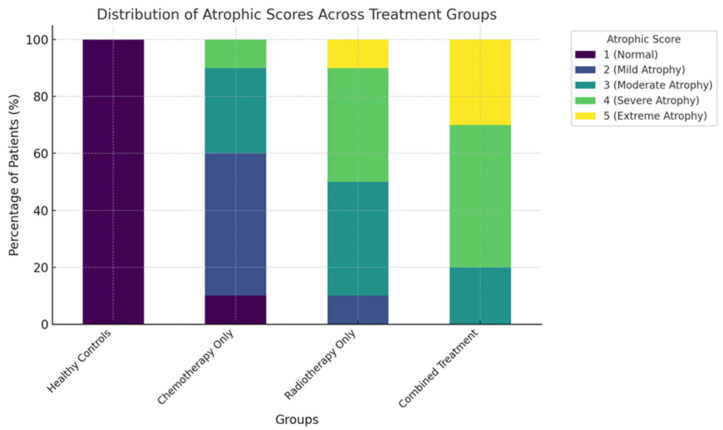
Distribution of Atrophic Scores Across Cancer Patients and Healthy Controls. This figure illustrates the distribution of atrophic scores for fungiform and filiform papillae in cancer patients (post-radiotherapy and chemotherapy) compared to healthy controls. The graph highlights the prevalence of moderate to severe atrophy (Scores 3–5) in cancer patients, particularly those undergoing radiotherapy, contrasted with the consistently normal scores (Score 1) observed in healthy controls. The visual underscores the significant morphological impact of cancer treatments on tongue papillae.

Filiform papillae are non-gustatory, and OCT demonstrated structural changes consistent with reduced filiform projections and altered reflectivity in the cancer cohort. However, we did not directly measure oral comfort, texture perception, mucosal injury, or secondary oral complications; therefore, any broader functional implications of filiform changes remain speculative and are not concluded from the present data. Figure 4 shows the distribution of fungiform and filiform atrophy grades across cancer patients and healthy controls, complementing Figure 3 by summarising between-group differences in atrophy severity rather than the association with patient-reported outcomes.

The extent of papillary atrophy varied significantly between treatment modalities, with radiotherapy and combined chemo-radiotherapy producing more pronounced morphological changes than chemotherapy alone.

Among patients who received radiotherapy, the mean height of fungiform papillae was 200 ± 40 µm, compared to 300 ± 50 µm in those treated with chemotherapy alone (*p* < 0.001). Filiform papillae were similarly affected, with a mean height of 100 ± 30 µm in the radiotherapy group versus 200 ± 50 µm in the chemotherapy group (*p* < 0.001). These differences were also reflected in the ordinal atrophic scores: most radiotherapy patients scored 4 or above, while chemotherapy patients typically scored between 2 and 3.

The group that received combined chemotherapy and radiotherapy showed the most severe papillary atrophy. All patients in this subgroup received a Grade 5 atrophic score, and OCT imaging revealed completely flattened papillae with near-total loss of structural definition and severely reduced signal reflectivity. Patient-reported taste impact were reported by 100% of patients in this subgroup, which is consistent with greater patient-reported symptom burden after combined treatment; however, without detailed treatment-dose mapping to tongue subregions and without objective gustatory testing, a dose–response relationship cannot be inferred from these data.

These findings suggest that radiotherapy—particularly when combined with chemotherapy—has a disproportionately damaging effect on tongue papillary structures. This may be due to both direct mucosal toxicity and the compounded impact of systemic and local therapies on epithelial regeneration and vascular integrity.

## 4. Discussion

In this case–control study, OCT identified significant differences in tongue papilla morphology between head and neck cancer survivors and healthy controls. Fungiform papillae demonstrated reduced morphometric dimensions in the cancer group, with similar structural changes also observed in filiform papillae. Patient-reported taste impact (UW-QOL) was more prevalent in the cancer cohort and showed a strong unadjusted association with fungiform papillary atrophy; however, interpretation is constrained by age imbalance between cohorts and by the use of patient-reported outcomes.

Age is plausibly related to both papillary morphology and perceived taste impact, and therefore represents a major potential confounder in this dataset. Consistent with this, age adjustment attenuated the structure–symptom association, but it remained statistically significant. These findings suggest that age explains part, but not all, of the observed association and underscore the need for cautious interpretation of unadjusted correlations in cross-sectional case–control comparisons.

Interpretation is further constrained by the exclusive use of patient-reported taste outcomes. In sensitivity analysis, additional adjustment for xerostomia (UW-QOL saliva domain) further attenuated the association and rendered it non-significant, supporting the interpretation that perceived taste impact may partly reflect broader oral symptom burden rather than isolated gustatory performance. Accordingly, OCT-derived papillary measures should not be interpreted as surrogate markers of taste function in this study.

Taken together, the data indicate an association between fungiform papillary atrophy and worse patient-reported taste outcomes, while filiform changes appear to occur in parallel; because broader oral mucosal injury and oral comfort were not directly assessed, we avoid attributing functional relevance to filiform findings beyond describing these co-occurring structural changes.

The proposed ordinal grading system showed substantial interobserver agreement, and the inclusion of healthy controls provided a reference baseline for interpretation. These findings should be interpreted as associative; longitudinal and age-matched studies are now needed to confirm directionality and clinical utility in survivorship follow-up.

The morphological changes observed in this study align closely with previously reported effects of cancer therapy on the oral mucosa. The severe atrophy of fungiform and filiform papillae among radiotherapy patients supports the findings of Shinde et al., who reported a strong link between radiation exposure to the tongue and subsequent taste dysfunction [22]. Likewise, our identification of dose-dependent changes mirrors the results of Gaillard et al., who highlighted the impact of mucosal dryness and epithelial thinning as central contributors to post-radiotherapy complications [23].

The more moderate but consistent morphological changes observed in chemotherapy patients corroborate the mucotoxic profiles of agents such as 5-fluorouracil (5-FU) and cisplatin. Wang et al. have previously documented delayed mucosal regeneration associated with these agents [24], which is reflected in the intermediate atrophic scores and structural alterations seen in this study. These findings are also supported by Zhang et al. [25] and Fiwek et al. [26], who identified prolonged taste impairment following systemic chemotherapy.

In contrast to earlier studies, the present research employed Optical Coherence Tomography (OCT) to obtain high-resolution, in vivo visualisations of papillary structure, adding a novel diagnostic dimension. While past research often relied on subjective clinical inspection of the tongue surface, clinician-reported grading of mucositis/atrophy, and patient-reported symptom questionnaires rather than direct in vivo structural assessment or histological analysis, our approach builds on recent colleagues’ work [27], demonstrating that OCT can quantify and stage papillary atrophy in a reproducible manner. The inclusion of a healthy control group further strengthens the validity of these findings by providing a clear morphological benchmark.

Overall, while the detrimental effects of cancer therapies on oral tissues are well-documented, this study contributes meaningful advances in diagnostic methodology and morphological characterisation, with direct implications for functional assessment and patient care.

This study faced several methodological challenges that warrant careful consideration. First, while OCT offers a non-invasive, high-resolution method to visualise oral mucosal structures, it is not without limitations. The technique is sensitive to motion artefacts, and image interpretation can be influenced by operator skill, probe positioning, and tissue hydration status. Despite efforts to standardise acquisition, residual variability may have introduced minor inconsistencies in scan quality.

Second, there is no widely accepted staging system for papillary atrophy in this context. Our five-point ordinal grading scale was defined a priori by investigator consensus using established OCT structural descriptors and anchored to healthy control appearances; however, the absence of an external reference standard limits comparability across studies and precludes claims of external validation at this stage.

Third, although interobserver agreement was substantial (κ = 0.76 for fungiform; κ = 0.71 for filiform), any observer-based scoring carries an element of interpretive variability. Quantitative morphometrics were included to mitigate this limitation, but measurement error cannot be fully excluded.

Fourth, the case–control design and single-centre recruitment limit causal inference and generalisability. The sample size (n = 53) and heterogeneity within the cancer cohort (tumour sites, treatment regimens, and time since therapy) introduce potential confounding that was not fully measurable or adjustable.

Fifth, selection bias is possible because controls were recruited by advertisement and were younger than the cancer cohort, reflecting feasibility constraints. Because papillary morphology and taste-related outcomes may vary with age and general health status, incomplete age matching may have influenced between-group differences. Importantly, age-related variation may also contribute to the observed correlations between papillary measures and UW-QOL taste outcomes, meaning treatment-related effects cannot be separated from age effects in the present dataset.

In addition, taste dysfunction was assessed using patient-reported outcomes (UW-QOL) without objective gustatory testing; therefore, common-source reporting (shared-method) bias and co-occurring symptoms (e.g., xerostomia and olfactory changes) may influence perceived taste and could contribute to the observed OCT–taste associations. Accordingly, findings should be interpreted as associative and hypothesis-generating; future studies should include age-matched controls and incorporate objective taste testing and key treatment/dose covariates where feasible.

However, including a comparator group with xerostomia unrelated to cancer treatment—such as patients with medication-induced or autoimmune dry mouth—could have provided additional insights into whether papillary atrophy and taste changes are driven primarily by salivary dysfunction or are unique to oncological therapy. Future research should consider this refinement in study design to better disentangle causative factors.

In addition, taste perception is a multisensory experience that relies not only on tongue papillary morphology but also on olfactory input and the presence of saliva to solubilise tastants. Both olfactory function and salivary flow may be impaired in cancer patients due to radiotherapy or chemotherapy, introducing potential confounding variables. These factors were not independently measured in this study, limiting our ability to isolate papillary morphology as the sole contributor to taste dysfunction.

Finally, while the cross-sectional design allowed for morphological comparisons at a single time point, it did not permit analysis of temporal changes in papillary recovery or degeneration. Longitudinal data would be essential for assessing the progression of atrophy or recovery post-treatment and for evaluating the predictive value of OCT measurements over time.

Despite these limitations, the methodological framework established in this study—including the use of OCT and a reproducible scoring system—lays the groundwork for future, larger-scale investigations and highlights key areas for refinement in oral mucosal imaging.

While a strong association was found between papillary atrophy and taste disturbance, the directionality of this relationship remains unclear; both may be concurrent outcomes of treatment-induced epithelial and salivary gland damage rather than a direct cause-effect pathway.

These findings support the feasibility of OCT for non-invasive, in vivo characterisation of tongue papillary morphology in head and neck cancer survivorship. OCT provides high-resolution imaging of papillary architecture and enables reproducible description of treatment-associated structural changes. However, given the cross-sectional design and reliance on patient-reported outcomes, the present data do not support predictive, diagnostic, or causal claims regarding taste outcomes.

Although fungiform atrophy was associated with worse UW-QOL taste scores, OCT-derived metrics cannot be considered surrogate markers of functional impairment in this study because outcomes were self-reported and objective gustatory testing was not performed. Any clinical implications should therefore be framed as hypothesis-generating. Future longitudinal studies incorporating objective taste testing, olfaction assessment, and salivary flow measures are needed before OCT-derived metrics can inform supportive intervention pathways.

Furthermore, the reproducible atrophy scoring system provides a standardised method for describing papillary morphology on OCT and may facilitate consistency across future studies. Its value for monitoring progression, predicting symptom trajectories, or guiding follow-up requires longitudinal validation in age-matched cohorts with objective taste testing.

Taste impact after head and neck cancer therapy is frequently under-recognised despite its relevance to nutrition and quality of life. OCT-based morphological assessment may help document papillary changes systematically for research and phenotyping; however, whether such imaging improves symptom recognition or management outcomes remains to be established.

Overall, these findings highlight cross-sectional associations between papillary morphology and patient-reported taste impact and motivate longitudinal, age-matched studies to determine temporal relationships and potential clinical value.

This study establishes a foundation for future investigations into the morphological and functional consequences of cancer therapy on the tongue. Expanding the sample size and including patients from multiple centres with diverse cancer types and treatment regimens would improve the generalisability of findings and allow for more nuanced subgroup analyses [2,5,8,22].

A key next step involves designing longitudinal studies to monitor the temporal progression of papillary atrophy and potential recovery following the completion of cancer treatment. Such studies could clarify the temporal relationship between papillary morphology and taste-related outcomes and determine whether OCT measures have prognostic value for symptom persistence or resolution [5,9,11,27].

Technological advancements in OCT should also be explored. Portable OCT devices, improved image resolution, and the incorporation of automated image analysis algorithms could enhance clinical utility and reduce inter-observer variability. Emerging approaches in machine learning may allow automated classification of papillary atrophy grades and correlation with symptom profiles, thereby streamlining both diagnosis and monitoring [7,12,15,18].

Additionally, therapeutic innovation is warranted. Investigating pharmacological agents, dietary supplements, or physical therapies that preserve or regenerate papillary structure may mitigate the impact of cancer therapies on oral health. There is also scope for exploring barrier-protective agents, such as mucosal coatings or growth factor-rich gels, that can shield the dorsal tongue from the mucotoxic effects of radiation and chemotherapy [1,4,6,14,15].

Finally, multi-centre, age-matched longitudinal studies could evaluate whether combining OCT with objective taste testing improves understanding of survivorship outcomes and supports reproducible phenotyping of oral toxicity. Any implementation frameworks or staging systems should be developed only after validation against objective gustatory measures and clinically meaningful endpoints [8,9,18,22].

## 5. Conclusions

This exploratory, single-centre case–control study demonstrates that OCT can reproducibly characterise lingual papillary morphology and that greater papillary atrophy is associated with worse patient-reported taste impact after head and neck cancer therapy. However, age imbalance between cohorts, reliance on self-reported outcomes, and the cross-sectional design limit functional and causal inference. Age adjustment attenuated the structure–symptom association, and additional adjustment for xerostomia further reduced the association, suggesting overlap with broader oral symptom burden. Larger, age-matched longitudinal studies incorporating objective gustatory testing and subregional treatment-dose mapping are required to determine temporal relationships and clinical utility.

## Figures and Tables

**Table 1:** Descriptive Comparison of Papillary Morphology and Functional Outcomes.

Papilla Type	Atrophic Score *	Common in Cancer Patients	Observed in Healthy Controls	Morphology Description	OCT Signal Intensity	Patient-Reported Taste Impact (%) **
Fungiform	1 (Normal)	Rare	Common	Clear dome/mushroom shape	High reflectivity	0
Fungiform	2 (Mild)	Chemotherapy	Absent	Slightly flattened	Moderate (CT high, ET high)	25
Fungiform	3 (Moderate)	Radiotherapy	Absent	Flattened, less vascular	Reduced in CT	50
Fungiform	4 (Severe)	High-dose Radiotherapy	Absent	Nearly flat, severe depletion	Low in both ET and CT	75
Fungiform	5 (Extreme)	Combined Therapy	Absent	Completely flat/lost	None detectable	90
Filiform	1 (Normal)	Rare	Common	Pointed, uniform	High in keratin	0
Filiform	2 (Mild)	Chemotherapy	Absent	Rounded, reduced projections	Moderate in epithelium	15
Filiform	3 (Moderate)	Radiotherapy	Absent	Flattened, minimal projections	Low in epithelium	35
Filiform	4 (Severe)	High-dose Radiotherapy	Absent	Stub-like remnants	Very low	55
Filiform	5 (Extreme)	Combined Therapy	Absent	Structure diminished	None detectable	70

* A trophic score categories and associated OCT morphological descriptors (including papilla height, epithelial thinning, and internal reflectivity/signal intensity patterns) were defined a priori by investigator consensus, informed by established descriptions used in in vivo OCT assessment of oral mucosa and by published characterisations of lingual papillae morphology [33,34,35,37]. ** For filiform papillae, taste disturbance (%) reflects co-occurring UW-QOL taste-domain impairment within each atrophy grade and is not intended to imply a direct taste–structure relationship.

**Table 2:** Statistical Validation of Papillary Metrics and Functional Differences.

Comparison	Cancer Patients vs. Healthy Controls	95% CI *	*p*-Value *	Effect Size (Cohen’s d) **	Observer Agreement (Kappa)
Fungiform Height	−350 µm	[−380, −320]	<0.001	1.45	0.76
Fungiform Width	−150 µm	[−180, −120]	<0.001	1.20	0.76
Fungiform Signal Intensity	Moderate to Low vs. High	N/A	<0.001	N/A	0.76
Patient-Reported Taste Impact	65%	[+50, +80]	<0.001	N/A	0.76
Filiform Height	−250 µm	[−280, −220]	<0.001	1.30	0.71
Filiform Width	−80 µm	[−100, −60]	<0.001	0.90	0.71
Filiform Signal Intensity	Low vs. High	N/A	<0.001	N/A	0.71

* Continuous morphometric outcomes (papilla height and width) were compared between cancer patients and healthy controls using independent-samples *t*-tests (two-sided), with 95% confidence intervals for mean differences. Where comparisons involved more than two groups (radiotherapy only, chemotherapy only, and combined therapy), one-way ANOVA with Tukey post hoc testing was applied as described in Section 2.10. Categorical outcomes (e.g., OCT signal intensity category) were compared using the chi-square test (or Fisher’s exact test when expected cell counts were <5). The prevalence of taste disturbance is presented as a proportion derived from the UW-QOL (v4) taste domain, with confidence intervals calculated using the Wilson method. ** Interobserver agreement for ordinal grading was assessed using Cohen’s kappa (κ). Associations between UW-QOL taste outcomes and ordinal/continuous papillary measures were assessed using Spearman’s rank correlation coefficient (ρ).

## Data Availability

All data were anonymised at the point of collection and stored securely on pass-word-protected institutional servers.
